# Heteroatom-doped highly porous carbon from human urine

**DOI:** 10.1038/srep05221

**Published:** 2014-06-09

**Authors:** Nitin Kaduba Chaudhari, Min Young Song, Jong-Sung Yu

**Affiliations:** 1 Department of Advanced Materials Chemistry, Korea University, 2511 Sejong-ro, Sejong 339-700, Republic of Korea

## Abstract

Human urine, otherwise potentially polluting waste, is an universal unused resource in organic form disposed by the human body. We present for the first time “proof of concept” of a convenient, perhaps economically beneficial, and innovative template-free route to synthesize highly porous carbon containing heteroatoms such as N, S, Si, and P from human urine waste as a single precursor for carbon and multiple heteroatoms. High porosity is created through removal of inherently-present salt particles in as-prepared “Urine Carbon” (URC), and multiple heteroatoms are naturally doped into the carbon, making it unnecessary to employ troublesome expensive pore-generating templates as well as extra costly heteroatom-containing organic precursors. Additionally, isolation of rock salts is an extra bonus of present work. The technique is simple, but successful, offering naturally doped conductive hierarchical porous URC, which leads to superior electrocatalytic ORR activity comparable to state of the art Pt/C catalyst along with much improved durability and methanol tolerance, demonstrating that the URC can be a promising alternative to costly Pt-based electrocatalyst for ORR. The ORR activity can be addressed in terms of heteroatom doping, surface properties and electrical conductivity of the carbon framework.

Nanostructured carbon materials are recognized as one of the most promising candidates for advanced energy applications of future generations, which require excellent structural and functional properties^1^,^2^,^3^. Carbon nanostructures have been widely studied as support material of various electrocatalysts for fuel cell^4^,^5^. Fuel cell, a sustainable energy conversion device for power generation, usually employs platinum and its alloys as the electrocatalyst for its redox reactions^6^,^7^,^8^. However, low natural availability of precious Pt metal catalyst and its cost implications are one of the key concerns in the commercialization of fuel cells^9^,^10^. Thus, investigation of precious metal-free catalysts which compete equivalently with Pt in terms of activity and stability is a major focus of the research interest worldwide^11^,^12^,^13^,^14^. In recent years, remarkable progress on heteroatom doping in the carbon framework has been persistent to develop various metal-free doped carbon that can potentially substitute the precious metal^15^,^16^,^17^,^18^,^19^,^20^,^21^,^22^,^23^,^24^. In particular, various carbon nanostructures such as CNTs, graphene, and porous carbons doped with various heteroatoms have been prepared and utilized as electrocatalysts for oxygen reduction reaction (ORR) in fuel cells^25^,^26^,^27^.

The development of simple and cost-effective strategies to synthesize carbon-based materials that have excellent structural and functional properties is one of the foremost challenges in materials chemistry at present^23^,^28^. In addition, to find a scale-up industrial method for synthesizing highly porous carbon is always challenging. So far, much effort has been devoted for the synthesis and development of various carbon materials with tailored properties using sacrificial inorganic hard templates, number of polymer/polymer precursors-based soft templates, various fossil/non-fossil-based materials, and other miscellaneous techniques^2^,^29^,^30^,^31^. In spite of their advantages, majority of the traditional synthesis routes for the porous carbon material typically involve multi-step procedures. These processes include, first, synthesis of various hard/soft templates followed by the carbon precursor's infiltration/impregnation, then cross-linking and high temperature carbonization of the precursors and finally template removal *via* strong alkali, acidic or caustic dissolution of the inorganic matrix^32^. These negative aspects on cost, environment, time-consuming and complex routes are seriously limiting the development of nanostructured carbon materials at the industrial scale. Fossil-based carbon sources are non-renewable and also need further processes including activation^33^. In addition, a variety of natural biomass-based materials, derived from plants and animals, have been transformed into the carbons via critical steps followed by post activations^34^,^35^,^36^,^37^,^38^. Synthesis from these sources not only produces significant carbon materials, but also often enables various doped heteroatoms, which are believed to be responsible for the improvement in activity towards various electrochemical reactions. Since high specific surface area is regarded as a primary requirement for the carbon materials, various activation procedures (e.g. physical and chemical activation by KOH, NaOH, ZnCl_2_, CO_2_, steam, etc.)^33^, which are usually highly corrosive and energy-intensive, are needed in most of the cases to develop and tailor the microstructures of carbon materials. Therefore, such synthesis routes are not only complex, but also resource and process-intensive and ultimately expensive from an industrial viewpoint. As a result, worldwide researchers are still struggling to find green and simple ways to obtain highly porous carbonaceous materials in sufficient quantities at a relatively low cost to sustain commercial requirements. Therefore, if such porous carbon materials can be prepared through a simple efficient methodology employing renewable sustainable abundant carbon precursor, this would mark major progress in the future.

Here, we present a ‘proof of concept’ that urine, which is one of the most abundant wastes on earth, can become the “carbon material of the future”. Human urine, otherwise potentially polluting waste, is an universal unused resource in organic form disposed by the human body. In current findings, the human urine is utilized to produce novel carbon, “Urine Carbon (URC)” for present and future energy related applications. According to analysis, the largest constituent of urine is urea, which is a significant organic source of H, C, O, and N^39^. Human urine is a complex aqueous solution; the other major inherent components present in the urine are P, S, Si, Na, K, Mg, Cl, and other minor traces. Tyrosine *O*-sulphate, a protein residue, and thiosulfate are daily excreted in considerable amounts in human urine which are key source of S^40^. Similarly, Si and P complexes are also eliminated into urine from human body through various metabolic processes^41^,^42^. Thus, the presence of these elements makes urine as a vital precursor for the synthesis of distinctively doped carbon. The practice of using human waste, i.e. urine as a fertilizer is as old as humanity itself. However, despite being used to produce hydrogen via urea and fed for the microbial fuel cells^43^,^44^,^45^, till date no report is available where human liquid waste i.e. urine was used as a precursor to produce carbon or any other materials. Judicious utilization of urine as carbon precursor will not only improve cost-effectiveness in development of functional materials, but also help to reduce the environmental burden by preventing the nitrogen, potassium, and phosphate-rich urine pollutants from reaching the water bodies, thus minimizing the formation of algal blooms, oxygen-robbing eutrophication and formation of oceanic “dead zones” in rivers and oceans^46^. Moreover, human body does not metabolize pharmaceutical drugs totally and expel some intact or in conjugated form along with other organic contaminants in urine. These compounds are carried by domestic waste streams to municipal wastewater treatment plants, or in some cases, directly to receiving water without treatment^47^, posing several environmental concerns. In this finding, converting urine to carbon will not only alleviate much of the above environmental concerns, but also assist in developing valuable materials for various technical applications including fuel cell, supercapacitor, Li-ion battery, and many more to mention, which can make our technique financially, technically, and environmentally favourable.

## Results

**Figure 1:**
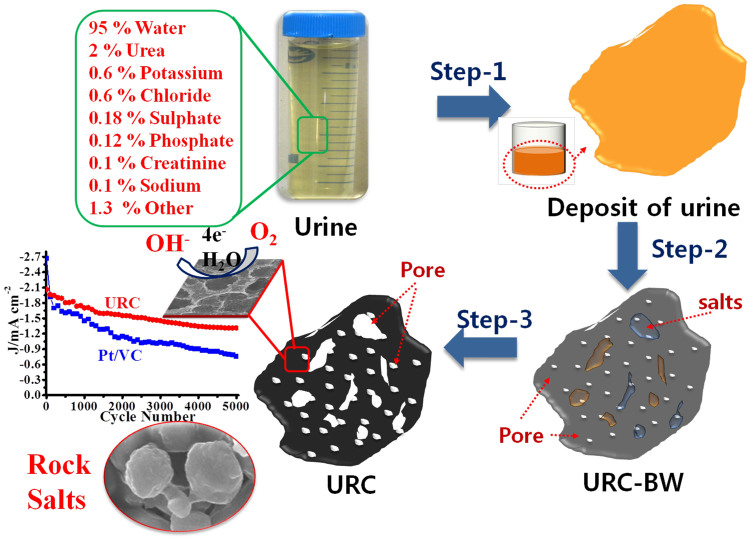
Schematic depiction of porous URC from human urine. Step-1: a dried yellowish brown deposit of urine obtained at 80°C for 48 h. Step-2: carbonization of the deposit under N_2_ flow for 6 h. Step-3: etching and washing by 0.1 M HCl to remove the rock salts mixture that generates various pores within the carbon structure.

**Figure 2:**
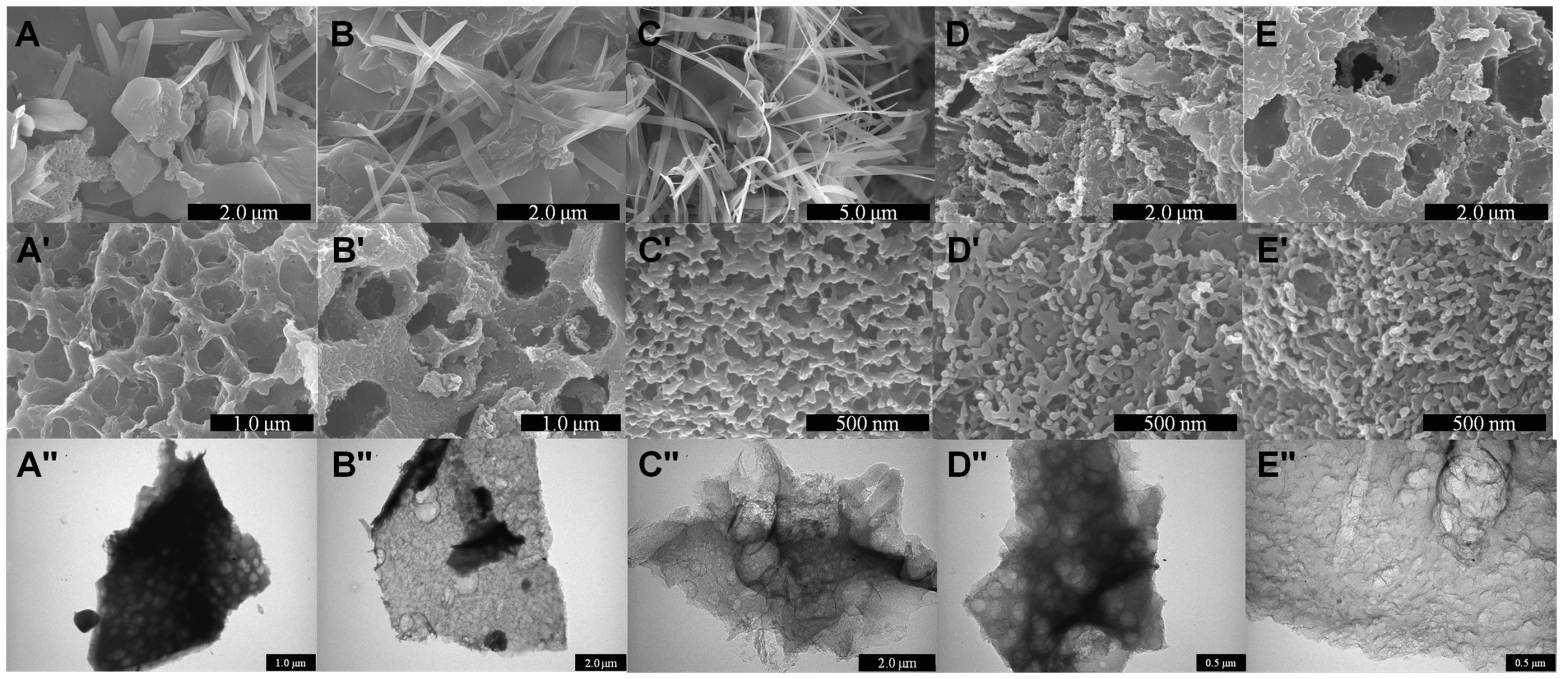
Effect of carbonization temperature on the formation of porous carbon from human urine. SEM images of mixture of carbon and rock salts obtained at URC-X-BW, where X is (A) 700, (B) 800, (C) 900, (D) 1000, and (E) 1100 before washing with diluted HCl. Whereas, (A′) SEM and (A″) TEM images of URC-700, (B′) SEM and (B″) TEM images of URC-800, (C′) SEM and (C″) TEM images of URC-900, (D′) SEM and (D″) TEM images of URC-1000, and (E′) SEM and (E″) TEM images of URC-1100 after HCl washing, demonstrating the porous morphology of carbon obtained after the HCl treatment.

To explore the potency of our projected assumption: human urine to carbon, we carbonized the dried yellowish deposit of the urine to 700 through 1100°C for 6 h in inert atmosphere to systematically analyze the effect of carbonization temperature on characteristics of resulting carbon (see Method Section). The detailed schematic depiction for the synthesis of the porous URC is shown in Figure 1. The water was fully removed from the collected urine samples by heating at 80°C for 48 h during the step-1 to obtain dried yellowish deposit from urine prior to the carbonization as shown in schematic depiction in the Figure 1. The obtained carbon powder is grayish black and from scanning electron microscopy (SEM) images, appears as a mixture of micro-size particles and nanofibrous structure as shown in Figure 2A–E. X-ray diffraction (XRD) measurements of these materials reveal rather complex signals of carbonaceous material and mixture of rock salts such as Sylvite and Halite, which are mineral forms of KCl and NaCl, respectively (Figure S1A in Supplementary Information (SI)). Interestingly, the XRD signal intensity of Sylvite gradually decreases compare to that of Halite as carbonization temperature increases and almost disappear after 1000°C. As the carbonization temperature increases beyond 900°C, the salts start to gasify by sublimation process and deposit as white powder on the inner wall of quartz tube inserted into the tube furnace, which is determined as mainly the evaporated Sylvite by XRD analysis. Since human urine contains as major constituents urea, chloride, sodium, potassium, creatinine, and other inorganic and organic compounds and ions^28^, the urine is expected to result in a composite consisting of carbon and mixture of salts upon carbonization. To obtain pure carbon material, the mixture of rock salts (Sylvite and Halite) and other minor inorganic compounds present were removed by treatment with 0.1 M HCl solution. The rock salts as particles dispersed throughout the carbon composite unwittingly leave behind small pores, i.e. meso/macropores as well as micropores on and within the carbon structure. Thus the inherently present rock salts serve as natural porogen for the formation of beneficial pores in the carbon framework.

To understand the mechanism of URC formation, it is worth pointing out that the composite is composed of a mixture of micro-sized particles and fibrous materials prior to HCl washing as seen in SEM images of Figure 2A–E. Upon increasing the carbonization temperature, the micro-sized particles break down into smaller structures and once-predominant fibers almost disappear (Figure 2D–E). The micro-sized particles mainly belong to carbon and the fibrous structure to inorganic salts. During carbonization at temperatures over 900°C, the majority of the volatile inorganic salts present in the dried urine starts to gasify by sublimation and results in a weight loss, which is consistent with decay of fibrous materials. Thus, the yield of resulting carbon varies with temperature of carbonization due to the change in the content of volatile salt materials. As an example, the weight for URC-1100-BW sample is 60% less compared with that of URC-700-BW before washing by HCl. This sublimation process is prominent, and eventually the gasified salts cool down and are deposited on the inner wall of the tube furnace as white powder, which is found to be the mixture of rock salts by a range of analysis (Figure S2 in SI). The salts separated from carbon is an extra bonus of present work, which can be recovered from human waste and finds valuable applications such as de-icing salts. Thus, due to the presence of the salts in urine, numbers of pores are developed in the URC.

The SEM (Figure 2A′–E′) and TEM (Figure 2A″–E″) images of the URC clearly give an idea of the carbon having highly porous structure created by removal of salts. Consequently, we were able to obtain porous carbon material with higher surface area by carbonization and by just removing the inherent inorganic salts using dil. HCl. These results imply that the proposed synthesis route is very effective for developing pores and eventually increasing the surface area in URC materials without use of common troublesome pore-generating inorganic templates or post-activation^33^,^48^. In general, the urine deposit carbonized and separated from salts by acid treatment produces 300–400 mg of porous URC using 1 L of human urine in current simple template-free process; however, the final carbon yield is mainly dependent on the organic contents of the urine and carbonization temperature. Normally human adult excretes about 1 to 2 L of urine/day depending on health and age. Thus, from every healthy human's body, sterile liquid wastes can produce approximately 300–800 mg of URC per day. Moreover, urine can be freely and easily collected in large quantity form public toilet/bathrooms for the carbon synthesis, and drying or evaporating larger volume of urine in an open air or sunlight involves comparatively much lower cost, but needs sufficient space.

The as-obtained salt-free carbon was further characterized by XRD, thermogravimetric analysis (TGA) and Raman measurement, which gives more insight on carbon formation using human urine waste (See Figure S1 to S4 in SI). The XRD patterns (Figure S1B in SI) of the carbon materials clearly revealed the changes after washing by 0.1 M HCl to remove the inorganic salts and other impurities. The XRD patterns of all the URC samples reveal the characteristic broad maxima peaks around 2θ = 25 (002) and 44° (100) of typical turbostratic carbon structure, while almost no characteristic signals for the salts or other impurities were observed. The (100) reflection corresponds to the honeycomb structure which is formed by sp^2^ hybridized carbons, and tends to be more intense as carbonization temperature increases. On the other hand, the (002) reflections between 20 and 30°, corresponding to coherent and parallel stacking of graphene like-sheets, become broader, indicating the increase of amorphous nature in the URC samples as the temperature increases from 700 to 1100°C. The shifts towards the smaller angles for the (002) peak at higher temperature also suggest an increase in the interlayer spacing due to increased amorphous nature. This increase in the d-spacing is related to the doping of heteroatoms, which are located or incorporated into the graphite layers of the carbon materials. Moreover, the presence of heteroatoms, especially N and S within the carbon structure increases the defects and disorder^49^. These results are in well agreement with the Raman measurement shown in Figure S4 of SI.

Thermal stability of the carbon materials is also important in determining their end use for numerous applications. These URC materials show a good thermal stability as shown in Figure S3 in SI. Raman spectroscopy was used to investigate the structural changes induced by the presence of the heteroatoms in the URC material as shown in Figure S4 in SI. Raman spectrum of each of URC samples displays two broad bands at 1340 and 1575 cm^−1^, which are assigned to the D band and G band, respectively. The positions of these bands are similar to each other for all five URC materials, suggesting that the structures of the all carbon are similar, showing turbostratic feature as observed in XRD spectra of Figure S1B. Interestingly, however, the differences in the I_D_/I_G_ ratio are observed, and the increase in the intensity ratio suggests that the carbon structure becomes more disordered with increasing carbonization temperature.

**Figure 3:**
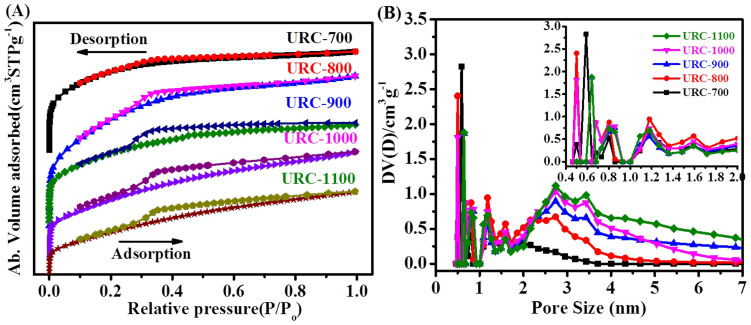
(A) Nitrogen adsorption–desorption isotherms and (B) the corresponding DFT pore-size distribution curves of the URC materials (Inset: magnified pore-size distribution).

Surface properties of the as-obtained carbon materials are very important for the electrochemical performance when used as an electrode material for ORR. Figure 3 reveals nitrogen isotherms and pore size distribution curves of the URC samples. Table 1 summarizes the total BET surface area, pore volume and pore diameter of the URC structures prepared at different temperatures. Porous structure can be generated by two main processes; first, the pores created by the evaporation of the salts present in the urine during carbonization process and secondly, the pores generated by dilute acid washing to remove the remaining salts particles from the carbon structures after the carbonization. N_2_ isotherms for all the URC carbons exhibit more or less similar type I isotherm typical of microporous carbon materials. The presence of a H4 hysteresis loop is indicative of a solid containing both micropores and mesopores. URC-700 shows a steady adsorption curve as the pressure increases with limited hysteresis loop, suggesting the presence of parallel or slit-like pores. While, as the carbonization temperature increases, clear hysteresis loop profile along with increase in the adsorption curve at higher pressure was observed. This could be due to the changes in the pores types from parallel or slit-like to cage-like pores. Additionally, in general, the micropore volume decreases from URC-700 to URC-1100. BET surface area is 1080.8 m^2^/g for URC-700 and increases to 1436.8 m^2^/g for URC-800, but then decreases to 1064.9 and 811.4 m^2^/g for URC-900 and URC-1000, respectively. Further increase in the carbonization temperature beyond 1000°C decreases the surface area greatly probably as a large portion of the salts has already evaporated, and this reduces the second step pore formation chance by acid washing. In particular, at 1000°C and higher, large portion of the inorganic salts present in initial dried urine deposit are evaporated and found on the inner wall out of heating zone of quartz tube due to the gasified sublimation (Figure S2B and D in SI), which is evidenced by greatly reduced weight of the carbon-salts composites and weak signal intensity of salt particles as shown in XRD results of URC-1000-BW and URC-1100-BW (Figure S1A in SI). The pore-size distribution calculated by density functional theory (DFT) method indicates that the carbonization temperature has some interesting effect on the average mesopore diameter of the URCs^50^. As the temperature increases from 700 to 1100°C, the mesopore diameter also increases from 2.1 nm (URC-700) to 3.2 nm (URC-1100) as presented in Table 1. Interestingly, URC-1000 and URC-1100 show similar mesopore diameter, which is found to be ca. 3.2 nm as shown in Figure 3B. This evidently supports that these mesopores are mainly created by the evaporation of salts during the carbonization at higher temperature rather than HCl washing. As shown in the inset of Figure 3B, it is clearly seen that URC materials contain number of micropores with diameters in the range of 0.4 to 1.6 nm formed mainly due to the removal of salts inherently present in urine by HCl washing. URC-700, -800 and -900 samples reveal high micropore volume percentage amounting to 82, 86, and 81% of respective total pore volume, whereas both URC-1000 and -1100 show decreased micropore volume percentage of 21% of respective total pore volume as shown in Table 1.

**Figure 4:**
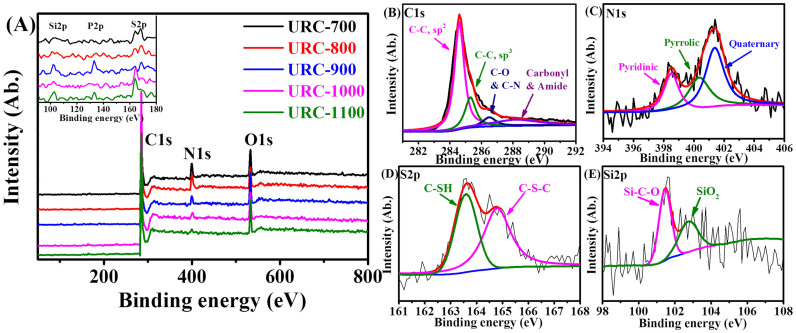
(A) XPS survey scan of URC prepared at various carbonization temperature. Inset: magnified XPS survey scan for Si, P, and S. and deconvoluted XPS spectra of (B) C 1s, (C) N 1s, (D) S 2p, and (E) Si 2p for URC-1000.

X-ray photoelectron spectroscopy (XPS) survey scan (Figure 4A) shows the presence of C, O, N, P, Si, and S in the URC. In particular, elements such as N, P, S, B and Si are widely believed to be responsible for the improvement in activity of carbon sample toward various electrochemical reactions^15^,^16^,^17^,^18^,^19^,^20^,^24^,^49^,^51^,^52^,^53^,^54^,^55^,^56^,^57^,^58^. Heteroatoms form covalent bond with the adjacent carbon in the carbon lattice, and therefore their catalytic degradation is much less as compared to that of Pt-based catalysts, which are usually generated through their physical attachment over the carbon support. Among them, nitrogen is by far the most investigated heteroatom because as a neighbor of carbon with different physicochemical properties, it is fairly easy to have N-doped carbon. Electronegativity of the nitrogen (3.04) and carbon (2.55) in the carbon matrix can destroy the electro-neutrality of the adjacent carbon. Moreover, the C-C bond length of sp^2^ hybridized carbon changes with the introduction of N. Because of the changes in bond length and electronegativity of carbon framework due to the N doping, carbon surface becomes asymmetric in nature and gets more active towards ORR^59^. Furthermore, N atoms doped at various active sites in the carbon framework play an important role in ORR due to their free lone pair electron available for the interaction with oxygen.

The elemental compositions are monitored to evaluate the changes in the chemical composition as a function of carbonization temperature and summarized in Table 1. The total N-content in the carbon decreased from 9.8 to 2.0% with increase in carbonization temperature. However, no significant temperature dependence was found on S content with its scanty content of 1%. Furthermore, Si and P were also present in trace amounts (less than 1%). Figure 4B–E shows the deconvoluted C 1s, N 1s, S 2p, and Si 2p photoelectron envelopes of URC-1000. C 1s spectrum indicates that the major fraction of carbon species is sp^2^ C at 284.6 eV, followed by sp^3^ (285.3 eV) hybridized C along with minor contribution (~286.5 eV) from different bonding configurations of carbon with oxygen or nitrogen (See Figure S5 of SI for details). Table S1 of SI summarizes the full width at half maximum (FWHM) values of sp^2^ and sp^3^ constituent peaks, which decrease by only 0.1 eV from URC-700 to URC-1100. This could be due to the effect of carbonization temperature and the presence of various heteroatoms in the URC. On the other hand, it is clearly seen that N 1s signal is split into three major peaks as pyridinic-N, pyrrolic-N, and quaternary-N (Figure S6 and S7 of SI). The FWHM values decrease distinctly for the deconvoluted constituent peaks for N1-pyridinic, N2-pyrrolic, and N3-quaternary configurations as shown in the Table S2 of SI. This may be attributed to the relatively higher nitrogen content (9.8%) for URC-700 compared to 2.0% in case of URC-1100. Sulfur is found to be present predominantly as two species such as aliphatic thiols or thioethers (~163.6 eV) and aromatic thiophenic sulfur (~164.6 eV). The Si 2p state of silicon is deconvoluted into two species such as Si-C-O (101.6 eV) and SiO_2_ (102.8 eV). The phosphorous content for URC-1000 was detected negligible (0.2%, see Table 1) in the survey scan and thus excluded from the deconvolution of P 2p peak.

**Figure 5:**
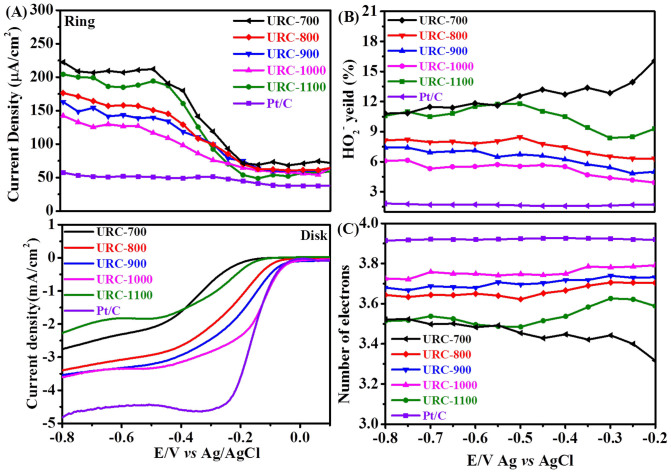
(A) Steady state ORR polarization plots of ring (top) and disk (bottom), (B) peroxide (HO_2_^−^) formation yield, (C) the number of electron exchanged during oxygen reduction at different potential for URC materials. The data of 20 wt% Pt/C (E-TEK) is also presented for comparison. (RDE/RRDE experiments were carried out at rotating speed 1600 rpm and 10 mV s^−1^ potential scan rate in O_2_-saturated 0.1 M KOH).

The cyclic voltammograms (CVs) at different carbon electrodes clearly show the oxygen reduction reaction (ORR) peaks near at −0.2 V *vs* Ag/AgCl in the O_2_-saturated 0.1 M KOH solution (Figure S8 in SI). The electrocatalytic activity and kinetic information of the various carbon materials for ORR were evaluated by rotating ring-disk electrode (RRDE) measurements and compared with commercial 20 wt% Pt/C in Figure 5. In Figure 5A, ring current decreases significantly from URC-700 to URC-1000, showing that the intermediate product of H_2_O_2_ was greatly decreased. The peroxide yield is less than 15% for all five URC materials at all potentials as seen in Figure 5B. The onset potential also shifts towards the positive direction, and the activity improves to greater extent from URC-700 to URC-1000 in alkaline solution as shown at the bottom of Figure 5A. The URC-1000 shows its onset potential (−0.03 V), almost identical to that of commercial 20 wt% Pt/C. The current densities are more or less −3.5 mA/cm^2^ (±0.01) for URC-800, -900, and -1000 electrodes, which are still less than −4.8 mA/cm^2^ for the 20 wt% Pt/C.

The *n* values for all five URC electrodes and Pt/C are shown in Figure 5C, which is about 3.7 ± 0.05 at different potentials for URC-800 to URC-1100. These results imply that oxygen reduction follows mainly an efficient four electron process similar to that of state of the art Pt/C electrode. The comparable catalytic activity of URC materials with commercial Pt/C has significantly important practical implications. Thus, the results confirm that the ORR activities of URC materials increased with the carbonization temperature from 700 to 1000°C. In particular, the URC-800, -900 and -1000 reveal excellent ORR performances with the best for URC-1000, whereas URC-700 and URC-1100 show comparatively poor ORR activity compared to the other three electrodes in accordance with CV results (Figure S8 of SI). This is attributed to insufficient carbonization and resulting high resistivity at low temperature of 700°C, and the comparatively low surface area and weak heteroatom content for URC-1100.

One of the major challenges in fuel cell applications is durability of electrocatalysts in the electrode^52^. In order to determine the stability of the URC materials, ORR forward peak maximum currents were recorded for URC-1000 and commercial 20 wt% Pt/C catalysts during the repeated potential cycling up to 5000 (Figure S9A of SI). After 5000 cycles, about 35% fading in activity was observed for URC-1000, whereas the Pt/C catalyst lost activity by more than 72%, indicating better long term stability for URC-1000. Pt is the best single electrocatalyst for oxygen reduction in a fuel cell. However, in an alcohol fuel cell, alcohol as fuel added in anode side can crossover the membrane toward the cathode electrode, where the alcohol can be oxidized, leading to loss of considerable activity. Therefore, methanol tolerance for the cathode catalysts is highly required to ensure a good performance. The CV scans for the commercial Pt catalyst in an electrolyte containing O_2_-saturated methanol (3.0 M) illustrate that the cathodic peaks for oxygen reduction disappear due to the more prominent methanol oxidation (Figure S9B in SI). However, in the same O_2_-saturated methanol solution (Figure S9C in SI), the URC-1000 maintains strong ORR selectivity with no specific activity to methanol oxidation, indicating that the URC-1000 catalyst is completely insensitive toward methanol. Obviously, the methanol oxidation on Pt/C caused by crossover of a high-concentration methanol fuel becomes very pronounced, which severely deteriorates the cell performance. In this study, the excellent methanol tolerance of the URC-1000 plays a key role, which maintains selective reduction of oxygen. This can give rise to a better cell performance in DMFCs and will be in fact potentially suitable for application to the passive-type DMFCs, which are designed to directly use high-concentration methanol as fuel.

**Figure 6:**
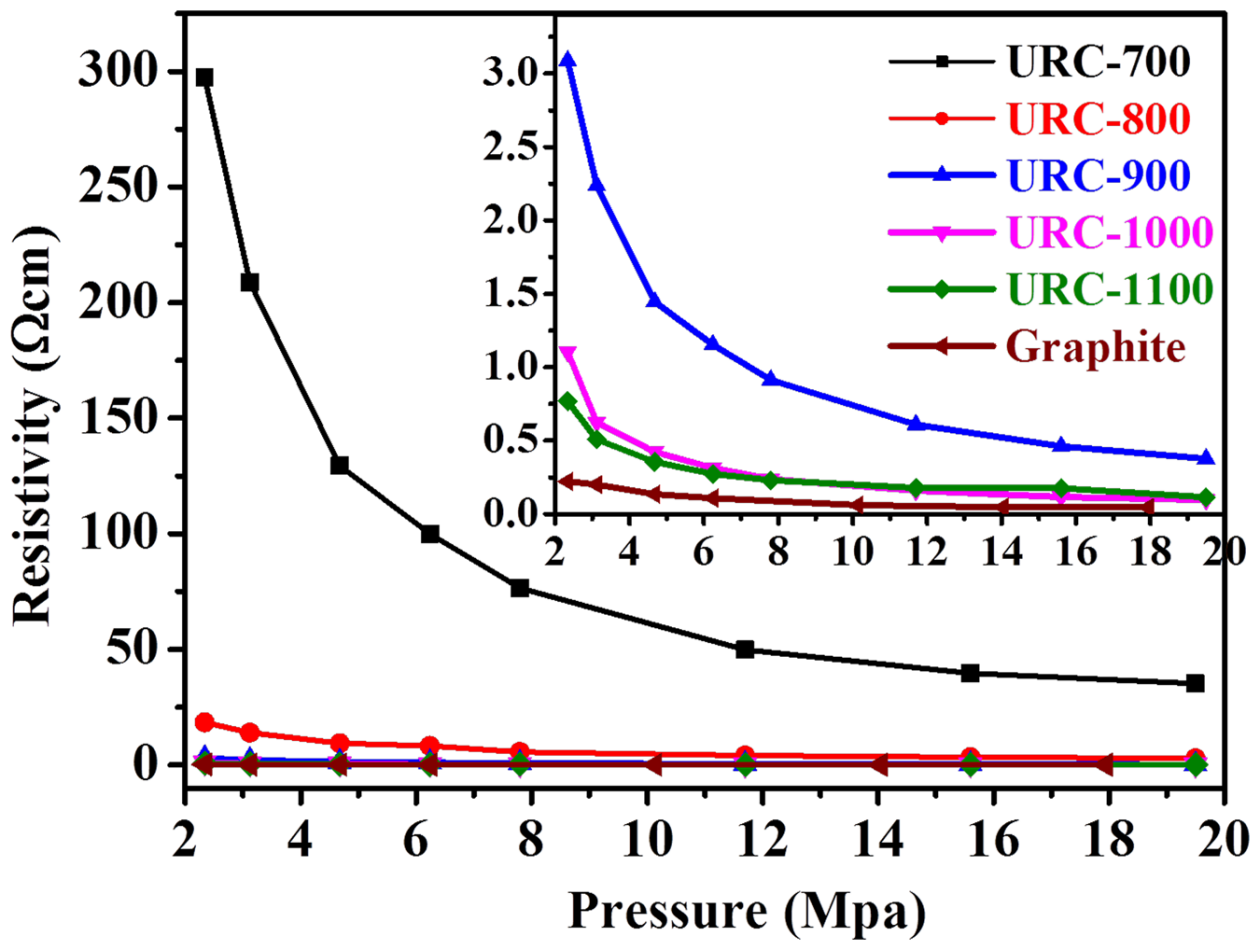
Resistivity *vs* pressure curves of the URC materials in comparison to graphite (Inset: magnified curves for URC-900, URC-1000, URC-1100, and Graphite).

In the investigation of electrode materials of electrochemical cells, the conductivity is also one of the essential properties of materials. The resistivity was measured as a function of applied pressure, using a resistance measuring device and a potentiometric circuit (See Figure S10 in SI for details). As shown in Figure 6, as the pressure increases, the resistivity decreases for all the samples including graphite. The URC carbon obtained at lower temperature (700°C) exhibits high resistivity, indicating the carbonization at higher than 800°C is favourable for electrical conductivity. This can be understood by the fact that the increase in temperature certainly improves the graphitization in agreement with the increase in sp^2^ hybridization (Figure S5 in SI), and eventually increases the overall conductivity. The URC-1000 and URC-1100 obtained in this work display conductivity similar to that of graphite.

Distribution of different major N species for URC materials prepared at different carbonization temperatures was determined from deconvoluted XPS spectra of N 1s as N is the most important for ORR (Figure S6 of SI). In general, while the total N content decreases with increasing temperature, the relative amount of the major N species shows interesting temperature dependency (Figure S7 of SI). While relative amount of pyrrolic N species mainly decreases rapidly with increasing temperature, pyridinic N species shows better stability with slow decrease. The relative amount of quaternary N species, which is known to be stable, increases with increasing temperature. All the N, S, P, and Si dopants in the URC materials are able to create defects on carbon surface and certainly contribute to the high ORR activity owing to charge accumulation in the doped materials^8^. In particular, nitrogen is known to enhance the electron-donor property of the carbon, which could lead to an increase the catalytic activity^23^. However, till date, the nature and the specific role of the catalytic centre of N-doped carbon have not been clearly understood. Even though there is no agreement in the literature regarding the role of different N species as ORR active sites, various studies believe that the pyridinic and pyrrolic-N species are responsible for the ORR activity^15^,^60^. It was also reported that the pyridinic-N among the different N species plays an important role as an active centre for ORR^15^. Recently, Lai et al. reported that not only pyridinic-N, but also quaternary-N aids as an active centre for the ORR^61^. The quaternary-N atoms in the carbon lattice can facilitate the electron transfer from the carbon electronic bands to the antibonding orbitals of O_2_ in ORR, improving the ORR activity because the electronic density of the N-doped carbon increases^62^,^63^. In light of these differences in the results, it is therefore reasonable to recognize that pyridinic-N and quaternary-N are the most active sites of N-doped metal-free ORR catalysts. From the RRDE results obtained from our URC work, we observe that URC-1000 sample shows more positive onset potential with higher current density. According to deconvoluted XPS spectra of N 1s as shown in Figure S6 and S7 of SI, the URC-1000 shows relatively higher percentage of quaternary-N along with pyidinic-N and small amount of pyrrolic-N compared to that of URC-700, -800, and -900. Hence there are strong possibilities that pyridinic-N and quaternary-N act as active centres for the ORR, which is also in good agreement with the general consensus.

## Discussion

Porous structure can be generated by two main processes; first by the evaporation of the salts present in the urine during carbonization process at high temperature and secondly by dilute acid washing to remove the remaining salts particles from the carbon structures after the carbonization. In addition, the carbonization temperature has some interesting effect on the development of micropores in the carbon. It is found that in general, micropores decreases with the increasing temperature as shown by the results of the micropore volume and the specific micropore surface areas (Table 1). On the basis of effect of carbonization temperature and pore volume determined by gas adsorption analyzer, the first pores created by the evaporation of the salts is likely to be mainly meso-macropores, while the second pores generated by HCl washing mainly correspond to micropores and macropores. In fact, higher mesopore surface area and pore volume are observed in URC-1000 and URC-1100 compared to those URC samples prepared at lower temperatures. This is mainly because a significant portion of the salts has already evaporated and this reduces the second step pore formation chance by acid washing at the carbonization temperature beyond 1000°C.

**Figure 7:**
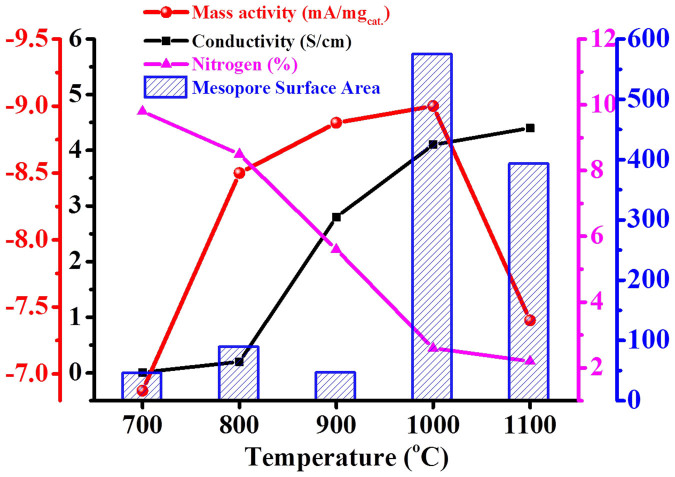
Comparative relation of electrical conductivity (at 8 Mpa), nitrogen content, and mesopore surface area along with calculated mass activities at −0.8 V *vs* Ag/AgCl of the URC materials as a function of carbonization temperature.

Figure 7 compares the surface area, total nitrogen content, and electrical conductivity (at 8 Mpa) along with ORR activity as a function of carbonization temperature. The mass activities (mA/mg_cat._) of the URC materials were calculated as −6.9, −8.5, −8.8, −9.0, and −7.4 for URC-700, 800, 900, 1000, and 1100 at −0.8 V *vs* Ag/AgCl, respectively. The URC samples prepared at 900 and 1000°C show 22 and 24% increased catalysts activity compare to that of the URC-700, respectively. The electrochemical activity of URC materials is strongly dependent on nitrogen content and the conductivity except for URC-1100. RRDE, XPS, Raman, resistivity and porosity data suggest that not only the catalytically active heteroatoms and electrical conductivity, but also textural and structural characteristics of the investigated carbon materials seem to be the key parameters that largely determine ORR performance. The better improved performance of the carbon electrodes obtained at 800–1000°C can be attributed to highly porous nature and good conductivity of the carbon along with proper amount of catalytically active N, S, P, and Si contents, which significantly enhance the ORR activity. It is a bit surprising that URC-1000, which has a lower surface area and also less N content than those of the URC-800 and URC-900, shows the overall best ORR performance. URC-1000 showed much lower resistivity than those of URC-800 and URC-900 samples, revealing better electrical conductivity.

Higher electrocatalytic activity of URC-1000 can be attributed to not only better electrical conductivity, but also higher mesopore surface area (more than 70%) and volume (near 80%), and increased interlayer spacing mentioned in XRD patterns of Figure S1B in SI, compared to those (low mesopore surface area and volume less than 6 and 20%, respectively) of URC-800 and URC-900 with microporous nature, which facilitates the O_2_ and electrolyte movement toward the ORR active surface sites. In fact, lower charge transfer resistances for URC-1000 and URC-900 than other URC samples are observed in electrochemical impedance spectroscopic plots (Figure S11 and Table S3 in SI). On the other hand, although URC-1100 is highly conductive, its poor ORR activity may be attributed to the other factors such as too low heteroatom content as well as low surface area. Based on the observations drawn from the results, the major issues of ORR can be addressed in terms of heteroatom content, electrical conductivity, and surface area, in particular mesopore surface area, which are the key factors governing the ORR activity (Figure 7). Among all the materials, URC-900 and URC-1000 were found to be the best materials. The URC-1000, despite of lower nitrogen content and total surface area compared to those of URC-900, possesses better electrical conductivity and higher mesopore surface area, which are critical for electron and mass transfer in fuel cell, and thus likely to surpass the URC-900 and others in terms of onset potential and current density.

In summary, we present for the first time a “proof of concept” of convenient and perhaps economically beneficial route to synthesize high surface area carbon containing heteroatoms such as N, S, Si, and P and high porosity good for electrochemical applications from human urine waste. Human urine, otherwise potentially polluting waste, is an universal unused resource in organic form disposed by the human body. Beneficial merits of URC include high porosity created through removal of inherently-present salt particles in as-prepared urine carbon in addition to doping of multiple heteroatoms into the carbon framework, making it unnecessary to employ troublesome expensive pore-generating templates as well as extra costly heteroatom-containing organic precursors. Additionally, isolation of rock salts is an extra bonus of present work, increasing additional value of the current approach. The current technique is simple, but very successful, offering naturally multiple-doped conductive hierarchical porous carbon, which leads to superior electrocatalytic oxygen reduction activity comparable to commercial state of the art Pt/C catalyst. Heteroatom content, high porosity for great surface area and electrical conductivity are found to be the major key factors governing the ORR activity. URC having optimized combination between doping content, surface area, and electrical conductivity reveals much improved ORR performance, which proves to be valuable in designing electrode materials for fuel cell. The findings should stimulate not only development of various novel carbon materials with superb functionality, but also extensive practical applications as electrochemical electrode materials and adsorbents. Thus, the URC is absolutely unprecedented and highly worthy material for making a stroke in fuel cell and other energy fields.

## Methods

### Synthesis of URC materials

The human urine samples were collected from healthy individuals with no prior medicinal examination in high-density polyethylene (HDPE) plastic bottle with wide mouth. Human urine contains urea (9.3 g/L), chloride (1.87 g/L), sodium (1.17 g/L), potassium (0.750 g/L), creatinine (0.670 g/L) as major constituents, and other inorganic and organic compounds and ions along with 95% water^28^. The average pH of the collected urine samples was around 7 to 7.5. To evaporate the water from the collected urine, the samples were dried in an oven at 80°C, resulting in the yellowish deposit; usually 100 ml of urine takes approximately 48 h to produce the yellowish deposit. The dried yellowish deposit of the urine was then transferred to a ceramic boat and carbonized in tube furnace under the N_2_ flow for 6 h by varying the carbonization temperature from 700 to 1100°C. Usually, temperature up to 600°C is considered to be too low for the carbonization along with poor electrical conductivity. In this regard, the carbonization temperature was varied from 700 to 1100°C to systematically analyze the effect of carbonization temperature on characteristics of resulting carbon. After the carbonization, the grayish black powder, namely URC-X-BW (X represents carbonization temperature, and BW signifies before washing), was dispersed in the beforehand prepared 0.1 M HCl solution (pH = 1.0), sonicated for 30 min to remove inorganic rock salts present in the as-prepared carbon, filtered under reduced pressure, and washed a few times with pure water to obtain a black carbon powder. The salt-removed black URC material was dried in oven at 80°C overnight, while the collected filtrate was dried to give the cream white powder of the rock salts. The yellowish urine deposit carbonized at 700°C followed by HCl treatment is labelled as URC-700, and correspondingly at 800, 900, 1000, and 1100°C as URC-800, URC-900, URC-1000, and URC-1100, respectively. Normally, 1 L urine produces 7 to 9 g of grayish black powder of carbon and mixture of salts, and finally 300–400 mg porous URC after removing inorganic rock salts or other inorganics present by HCl. Thus, this process generates not only highly porous heteroatoms-containing carbon with hierarchical architecture, but also useful inorganic rock salts. Details for surface and electrochemical characterizations are provided in the supporting Information. Our work does not include any experiment on live vertebrates and human subjects. Only waste urine disposed from our own body (authors) was collected and used as a precursor for synthesis of porous carbon with heteroatoms for ORR catalysis.

### Surface characterization

The scanning electron microscopy (SEM) images were obtained using a Hitachi S-4700 microscope operated at an acceleration voltage of 10 kV. The transmission electron microscope (TEM) was operated on EM 912 Omega at 120 kV. High-resolution scanning electron microscopy (HR-SEM) images were obtained on Hitachi S-5500 microscope operated at 30 kV. High-resolution transmission electron microscopy (HR-TEM) images were obtained on JEOL FE-2010 microscope operated at 200 kV. X-ray photoelectron spectroscopy (XPS) analyses were carried out with AXIS-NOVA (Kratos) X-ray photoelectron spectrometer using monochromated Al-Kα X-ray source (h&b.upsi; = 1486.6 eV) operated at 150 W under base pressure of 2.6 × 10^−9^ Torr. XPS spectra were deconvoluted using curve fitting program consisting of Shirley base line with a combination of 20% Gaussian and 80% Lorentzian function. XPS Peak-Fit 4.1 software was used for all data processing. X-ray diffraction (XRD) patterns were obtained using a Rigaku Smartlab diffractometer with CuKα radiation using a Ni β-filter at a scan rate of 2°/min. The X-ray source was operated at 40 kV and 30 mA. The nitrogen adsorption-desorption isotherms were measured at −196°C using a Micromeritics ASAP 2020 system. Specific surface areas of the samples were determined by nitrogen adsorption data in the relative pressure range from 0.05 to 0.2 using the Brunauer-Emmett-Teller (BET) equation. Total pore volumes were determined from the amount of gas adsorbed at the relative pressure of 0.99. Pore-size distributions were calculated using Micromeritics software based on density functional theory (DFT) method^50^. Thermal Gravimetric Analysis (TGA) was carried out on a Bruker TG-DTA3000SA thermal analyzer at a heating rate of 20°C/min in flowing air (60 mL/min), increasing from room temperature to 1000°C to investigate the decomposition course of materials. Raman spectroscopy measurements (Renishaw) were recorded using an Ar ion laser (λ = 514.5 nm). For the measurement of electrical conductivity of the URC materials, a special cell in four probe configuration was designed and constructed by our group for the measurement of the electrical resistance under controlled pressure. The electrical resistance is then measured using four probes under a fixed pressure, using Keithley model 6220 and 2182A as DC current source and voltmeter. The sample volume is calculated from the measured sample thickness and the area of the cross section of the pressure chamber.

### Electrochemical characterization

Electrochemical experiments were carried out at room temperature in a three electrode cell using rotating ring-disk electrode (RRDE) connected to an electrochemical analyzer (Biologic VMP3). The RRDE measurements were conducted with a fitted glassy carbon disk and platinum ring in an oxygen-saturated 0.1 M KOH. The carbon material or commercial Pt/C catalyst (5.0 mg) was dispersed in a mixture solution of water and 5 wt% Nafion (water:Nafion = 1:9). The as-prepared 10 μL catalyst ink was then dropped onto the glassy carbon (5 mm) of the RRDE (0.4 mg/cm^2^) and dried at room temperature to prepare working electrode. An Ag/AgCl with saturated KCl and a Pt wire were used as reference and counter electrode, respectively. The cyclic voltammetry experiments were conducted in O_2_-saturated 0.1 M KOH solution for oxygen reduction reaction (ORR) at the scan rate of 50 mV/s in the potential range from −1.2 to +0.3 V at room temperature. Rotating disk electrode (RDE) measurements were performed in the O_2_-saturated 0.1 M KOH solution at rotation speed of 1600 rpm with the scan rate of 10 mV/s from 0.2 to −0.8 V. For the methanol tolerance test, the ORR measurements in the absence and presence of 3.0 M methanol were performed in O_2_-saturated 0.1 M KOH at the scan rate of 50 mV/s in the potential range from −1.2 to +0.4 V at room temperature. For the long term cycling performance, the ORR forward peak maximum currents were recorded during the repeated potential cycling up to 5000 cycles at the scan rate of 50 mV/s in O_2_-saturated 0.1 M KOH solution.

The ORR electrochemical procedure of URC was performed using RRDE measurements. The hydrogen peroxide ions percentage (% HO_2_^−^) was calculated based on the following equation (1): 
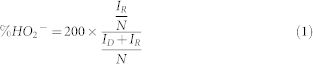
The electron transfer number (*n*) was determined from RRDE measurements on the basis of the disk current (*I_D_*) and ring current (*I_R_*) via the following equation 2: 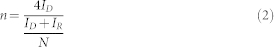
Where *I_D_* is the disk current, *I_R_* is the ring current, N is current collection efficiency of the employed Pt ring.

## Author Contributions

J.-S.Y. supervised and coordinated all aspects of the project. N.K.C. proposed and designed the experiment and performed the carbon synthesis and characterizations. M.Y.S. performed the electrochemical activity including RDE/RRDE tests and electron microscopy measurements. N.K.C. and J.-S.Y. wrote the article. All authors discussed the results and commented on the manuscript.

## Supplementary Material

Supplementary Information Supplementary Inforamtion

## Figures and Tables

**Table 1:** Atomic composition obtained from XPS spectra and physical characteristics by nitrogen sorption data for various URCs

	Atomic Composition (%)						Physical characteristics				
Sample	C 1s	O 1s	N 1s	S 2p_3/2_	Si 2p	P 2p	BET total surface area (m^2^ g^−1^)	Micropore surface area (m^2^ g^−1^)	Pore volume (cm^3^ g^−1^)	Micropore volume (cm^3^ g^−1^)	pore size (nm) by DFT method
URC-700	78.2	9.8	9.8	0.9	1.0	0.3	1080.8	1037.3	0.65	0.53	2.1
URC-800	81.9	7.7	8.5	0.9	0.7	0.3	1436.8	1349.7	0.83	0.71	2.6
URC-900	82.6	9.8	5.6	0.8	0.5	0.7	1064.9	1020.1	0.62	0.50	3.0
URC-1000	86.3	9.7	2.6	0.9	0.3	0.2	811.4	237.4	0.58	0.12	3.1
URC-1100	86.7	9.5	2.0	0.9	0.5	0.4	602.2	210.4	0.47	0.10	3.2
