# Supplementary material for: Heteroatom-doped highly porous carbon from human urine
Source: Sci Rep. 2014 Jun 9;4:5221. doi: 10.1038/srep05221 (PMC4049026; doi:10.1038/srep05221)
Supplement: Supplementary Information — Supplementary Inforamtion [file srep05221-s1.pdf]

Supplementary Information:

Heteroatom-doped highly porous carbon from human urine

Nitin Kaduba Chaudhari, Min Young Song & Jong-Sung Yu*

Department of Advanced Materials Chemistry, Korea University, 2511 Sejong-ro, Sejong 339-700, Republic of Korea.

*To whom correspondence should be addressed. E-mail: jsyu212@korea.ac.kr

X-ray diffraction (XRD) patterns for the URC

XRD patterns of carbon samples obtained from human urine before etching by diluted HCl are shown in Figure S1A. It is found that the material is a mixture of carbon and the rock salts, sylvite and halite, which are in good agreement with JCPDS-00-001-0790 (sylvite: KCl) and JCPDS-01-072-1688 (halite: NaCl). In cases of URC-1000-BW and URC-1100-BW, a wide peak around 25° is observed more clearly and can be attributed to the carbon, which is imperceptible for the samples obtained at lower carbonization temperature. This can be understood by the presence of more salts in the samples prepared at the low temperatures. For URC-1000-BW and URC-1100-BW, the XRD intensity was magnified three times for better clarification of the salt signals compare to those of low temperature samples.

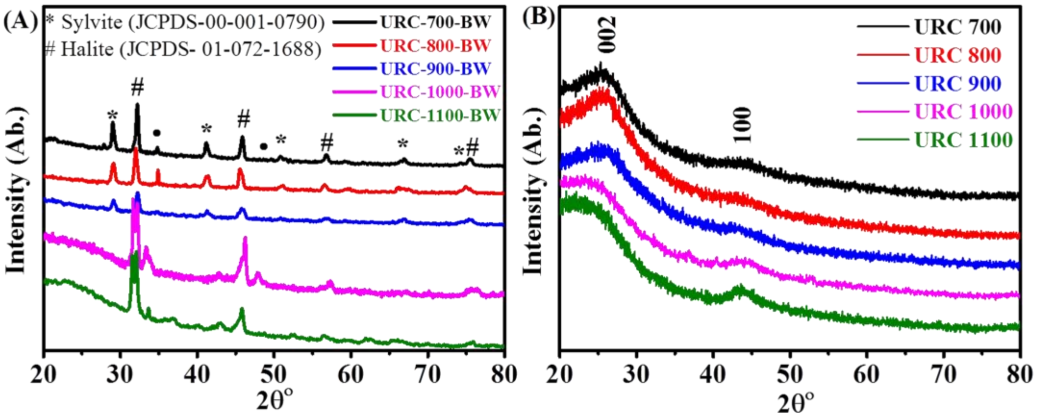

Figure S1 X-ray diffraction (XRD) patterns for the carbon samples obtained from human urine before (A) and after (B) etching by acid. ● indicates some other minor impurities. In (A), for URC-1000-BW and URC-1100-BW, the XRD signal intensity was magnified three times for better clarification of the salt signals compare to those of low temperature samples.

Both rock salt mixtures obtained from dried filtrate and evaporated deposit also consists of sylvites and halites, and some other minor salts are also present as shown in Figure S2B. From (a) of Figure S2B, interestingly, the dried rock salt mixture obtained as filtrate for URC-1000-BW reveals relatively much stronger halite peak intensity compared to sylvite. On the other hand, the evaporated salt deposit collected on the inner wall out of heating zone of quartz tube inserted into the tube furnace for URC-1000-BW shows much increased relative peak intensity of sylvite as seen in the XRD spectrum of (b), indicating prime sublimation of sylvites in the URC prepared at temperatures higher than 900 ^{o}C. (C) SEM image clearly shows the rod- or needle-like rock salt particles obtained by drying the filtrate with the average diameter of 180±50 nm and length in the range of 300 to 600 nm, which may be related to the development of fibrous materials as seen in Figure 2A-C in the main text, and (D) SEM image reveals evaporated salt particles found on the inner wall of quartz tube at 1000 ^{o}C. The salt particles are mainly found to be mixture of diverse shaped particles from cubic to hexagonal with size less than *ca*. 1 μm.

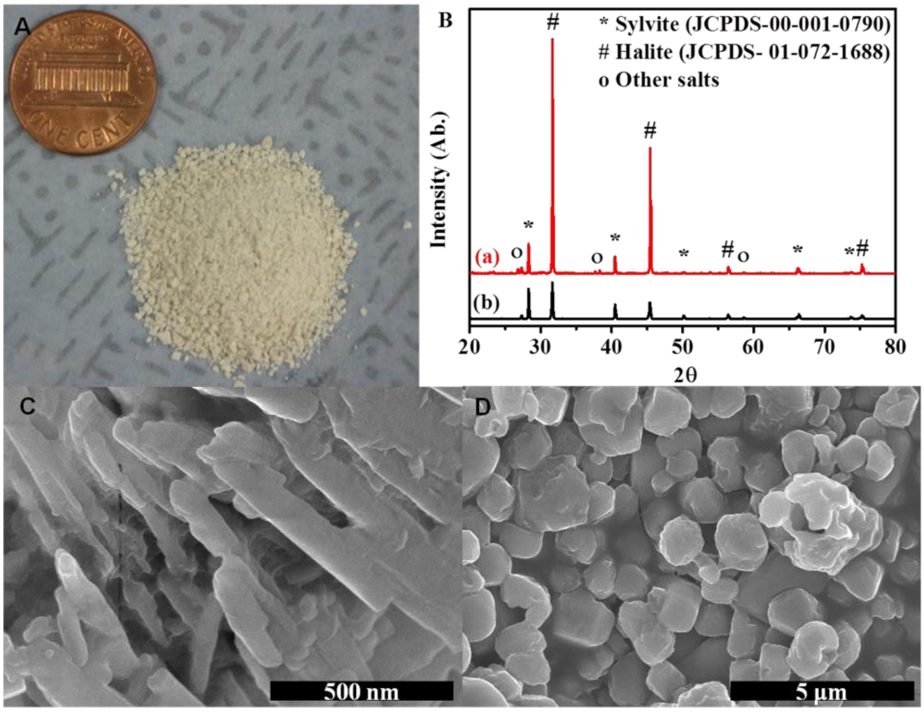

Figure S2 (A) A photograph showing rock salt composite and an USA one cent coin for comparison; The rock salt composite was obtained by drying the filtrate removed by 0.1 M HCl from the mixture of carbon and salt materials synthesized using human urine, (B) XRD spectra of (a) dried rock salt mixture (see (C) for the SEM image) obtained from filtrate for URC-1000-BW and (b) evaporated salt particles (see (D) for the SEM image) collected on the inner wall out of heating zone of quartz tube inserted into the tube furnace for URC-1000-BW.

Thermal stability of URC materials

The carbon content in these URC materials can be readily determined by TGA. As shown in the Figure S3, the weight losses near 100 ^{o}C are mainly attributed to the evaporation of adsorbed water. The combustion of carbon slowly begins around 460 ^{o}C for URC-700 and URC-800, and around 570 ^{o}C for URC-900, URC-1000, and URC-1100, which is comparable (565 ^{o}C) to that of graphite (Junsei Chemical Co., Ltd. Japan), indicating good thermal stability. The decomposition of URC samples is nearly complete after 700 ^{o}C. The final weight

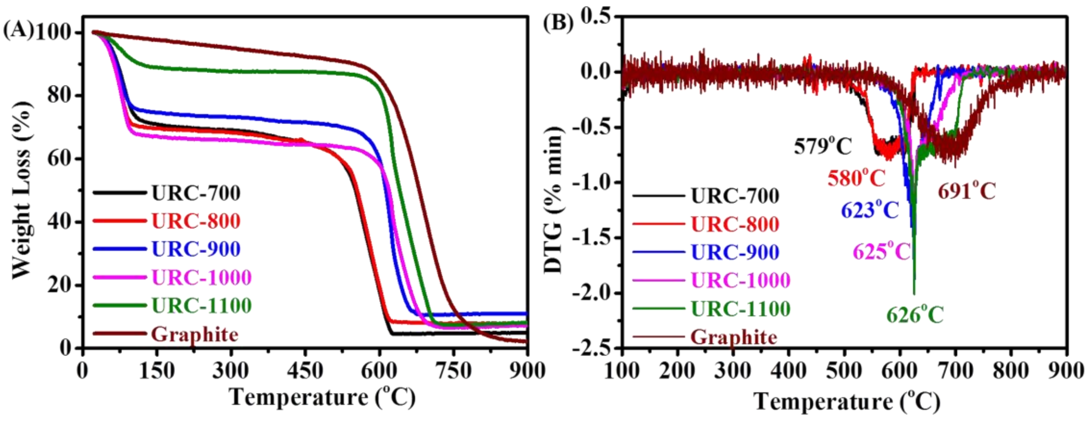

Figure S3 (A) Typical thermogravimetric analysis (TGA) plots representing weight percent *vs* temperature and (B) differential thermogravimetry (DTG) curves for the URC materials in comparison with graphite.

loss reaches to 95.5, 91.3, 89.6, 93.3, and 92.5 % for URC-700, URC-800, URC-900, URC-1000, and URC-1100, respectively. Secondly, thermal stability of the carbon materials is also important in determining their end use for numerous applications. These URC materials show a good thermal stability, and the peak temperature on DTG curve represents the temperature at which the maximum weight loss rate is reached, as shown in Figure S3B. According to the DTG curves, it is found that the ranges of decomposing temperature move toward higher temperature and significantly increased by about 47 ^{o}C from URC-700 to URC-1100, indicating that the increase in carbonization temperature considerably enhances the thermal stability of the URC materials.

Raman spectra of the URC materials

The presence of non-metallic heteroatoms such as S, Si, and P except N in all our samples is found comparatively low by XPS analysis techniques (e.g., each <1 atom %). Raman spectroscopy was used to investigate the structural changes induced by the presence of the heteroatoms in the URC material as shown in Figure S4. Raman spectrum of each of URC samples displays two broad bands at 1340 and 1575 cm^{-1}, which are assigned to the D band and G band, respectively. The D band at 1340 cm^{-1} is related to the breaking of symmetry caused by structural disorders and defects, while the G band at 1575 cm^{-1} represents the in-plane tangential stretch vibration mode (E_{2g}) of the graphite sheet (ref. S1, S2). Raman spectroscopy indicates that the peak intensities increase with the carbonization temperature. The positions of these bands are similar to each other for all five URC materials, suggesting that the structures of the all carbon are similar, showing turbostratic feature as observed in XRD spectra of Figure S1B. Interestingly, however, the differences in the I_D/I_G ratio are observed, and the increase in the intensity ratio suggests that the carbon structure becomes more disordered with increasing carbonization temperature. Moreover, it should be noted as well that at higher temperature i.e. for URC-1100, the second order D′ and G′ bands become more noticeable than for the other samples prepared at lower carbonization temperature. This can be attributed to the increase of the disorder in sp^2 hybridized graphitic

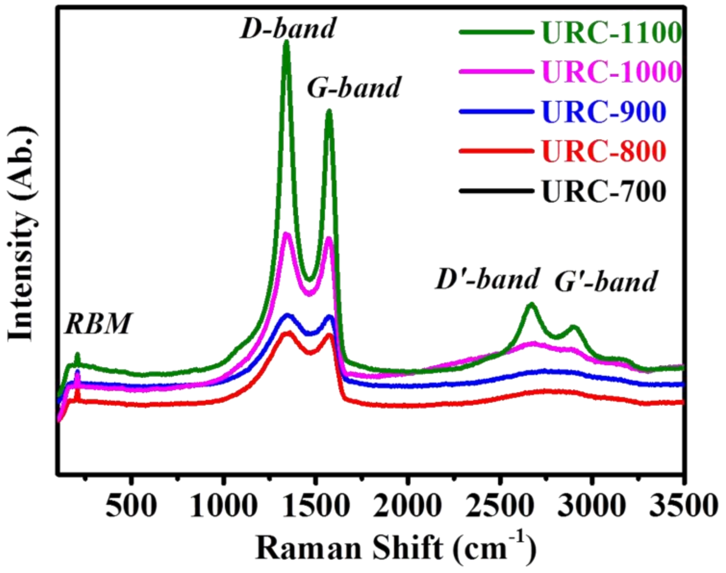

Figure S4 Raman spectra of the URC materials obtained at various carbonization temperatures showing RBM (radial breathing mode), D-band, G-band, D′-band, and G′-band regions.

carbon. The strong D- and D′-band peaks for URC-1100 indicate that defects are increasing in the carbon obtained at higher carbonization temperature owing to the various bonding structures and defects in the URC-1100. The G-band peaks of all the samples down-shifted to around 1575 cm^{-1}, compared to the pristine graphene peak at 1589 cm^{-1}, signifying the increase of defects and disorder due to the presence of the heteroatoms within the carbon structure. Nevertheless, the defects and disorder are considered to be favorable for the improvement of electrochemical properties (ref. S3).

XPS spectra of C 1s for URC materials

The major C 1s peak at 284.6 eV can be assigned to sp^2 (C-C) followed by sp^3 at 285.3 eV, and the peak at 286.5 eV corresponds to the bonding configurations of carbon with oxygen or nitrogen. (ref. S4-S8) As shown in Table 1, the carbon content increases from URC-700 to URC-1100. Similarly, the main peaks of C-C bonding i.e. sp^2–type (284.6 eV) and sp^3–type (285.3 eV) are also increasing along with the total carbon content (Figure S5). However, comparatively the increase in sp^2 hybridization is slightly higher over the sp^3 hybridization. Table S1 summarizes the full width at half maximum (FWHM) values of sp^2 (C-C) and sp^3 (C-C) constituent peaks. The FWHM values of sp^2 and sp^3 constituent peaks decrease by only 0.1 eV from URC-700 to URC-1100. This could be due to the effect of carbonization temperature and the presence of various heteroatoms in the URC. (ref. S9) Secondly, for the deconvoluation of the C 1s signal, the energies were chosen at 284.6 and 285.3 eV for sp^2 and sp^3, respectively.

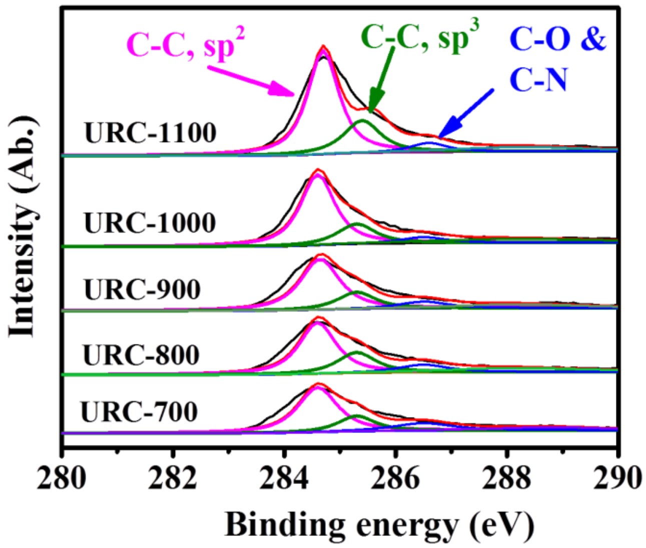

Figure S5 Deconvoluted XPS spectra of C 1s for URC materials prepared at different carbonization temperatures.

Table S1. FWHM values of deconvoluted peaks of C 1s in the URCs.

Peak	Sample	FWHM (eV)	
		sp^2 (C-C)	sp^3 (C-C)
**C1s**	URC-700	0.8	0.8
	URC-800	0.8	0.8
	URC-900	0.7	0.8
	URC-1000	0.7	0.8
	URC-1100	0.7	0.7

XPS spectra of N 1s for URC materials

Figure S6 shows the deconvoluted XPS spectra of N 1s for URC materials prepared at different carbonization temperatures. Figure S7 shows relative ratios of the deconvoluted peaks of N 1s as a function of carbonization temperature. It is clearly seen that N 1s signal is split into three major N species peaks centered at ~398.4 (pyridinic-N1), ~400 (pyrrolic-N2), and ~401.1 (quaternary-N3) (ref. S10). Interestingly, relative amount of N bonding configurations significantly changes with increasing carbonization temperature. It is worthy to note that all the N species decrease with the increase of temperature. Quaternary N species is found to be relatively more stable and predominant compared to pyridinic and pyrrolic N species although overall N content decreases with increasing temperature. In particular, pyrrolic N largely decreases probably owing to

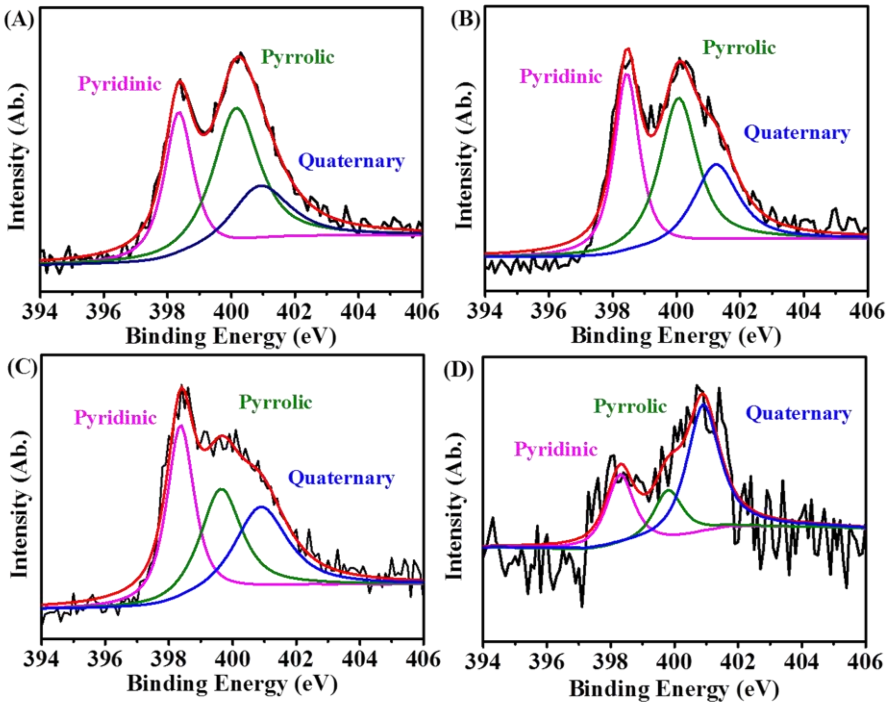

Figure S6 Deconvoluted XPS spectra of N 1s for URC materials prepared at (A) 700 °C, (B) 800 °C, (C) 900 °C, and (D) 1100 °C.

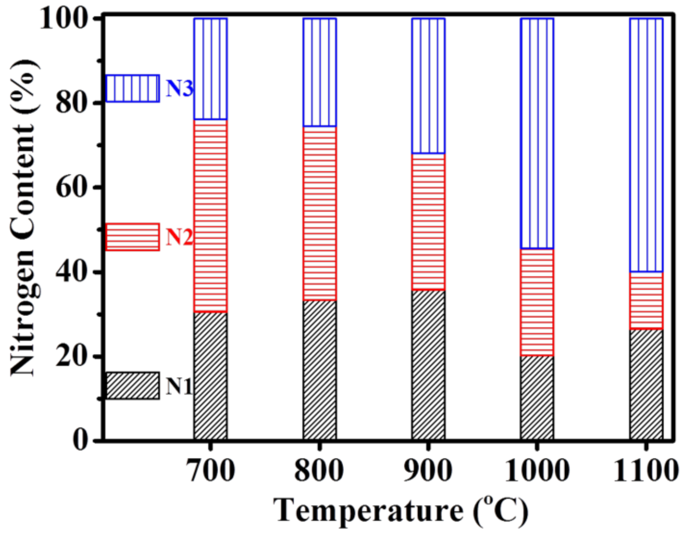

Figure S7 Relative ratios of the deconvoluted peaks of nitrogen. (N1: pyridinic, N2: pyrrolic, and N3: quaternary).

their lower stability at high temperature (Figure S7). Interestingly, pyridinic N species also decreases but to the less amount. Thus, the relative amount of the quaternary N species increases with increasing temperature probably due to relatively better stability of quaternary N species compared to pyridinic and pyrrolic N species. Moreover, all the URCs show presence of a minor terminal-N after 402 eV, which was excluded from the deconvoluted XPS spectra for clarity. In the case of XPS spectrum of N 1s, the FWHM values decrease distinctly for the deconvoluted constituent peaks for N1-pyridinic, N2-pyrrolic, and N3-quaternary configurations as shown in the Table S2. This may be attributed to the relatively higher nitrogen content (9.8 %) for URC-700 compared to 2.0 % in case of URC-1100 (ref. S7, S9). Furthermore, for the deconvoluation of the N 1s signal, the energies were chosen at 398.4 (pyridinic-N1), 400 (pyrrolic-N2), and 401.1 eV (quaternary-N3).

Table S2. FWHM values of deconvoluted peaks of N 1s in the URCs.

Peak	Sample	FWHM (eV)		
		N1	N2	N3
N1s	URC-700	1.1	1.7	2.2
	URC-800	1.0	1.6	2.0
	URC-900	1.0	1.4	1.7
	URC-1000	0.9	1.1	1.7
	URC-1100	0.9	1.0	1.5

Cyclic voltammogram (CV) measurements for the URC materials

In general, the onset potential of ORR and cathodic reduction peak increase with increasing carbonization temperature as shown in Figure S8. Compared with all the carbon electrodes, the URC-1000 electrode shows better and more positive shift in both the onset potential (-0.04 V) and the peak potential (-0.19 V) with additional notable increase in the current density. Interestingly, the ORR onset and cathodic peak potentials at the URC-700 and URC-1100 electrodes shifted negatively compared to those of the rest three carbon electrodes, indicating a reduced ORR electrocatalytic activity. In order to determine the stability of the URC materials, ORR forward peak maximum currents were recorded for URC-1000 and commercial 20 wt% Pt/C catalysts during the repeated potential cycling up to 5000 (Figure S9A). Methanol tolerance was compared for both URC-1000 and 20 wt% Pt/C catalysts in an electrolyte containing O_2-saturated 3.0 M methanol in Figure S9B and S9C.

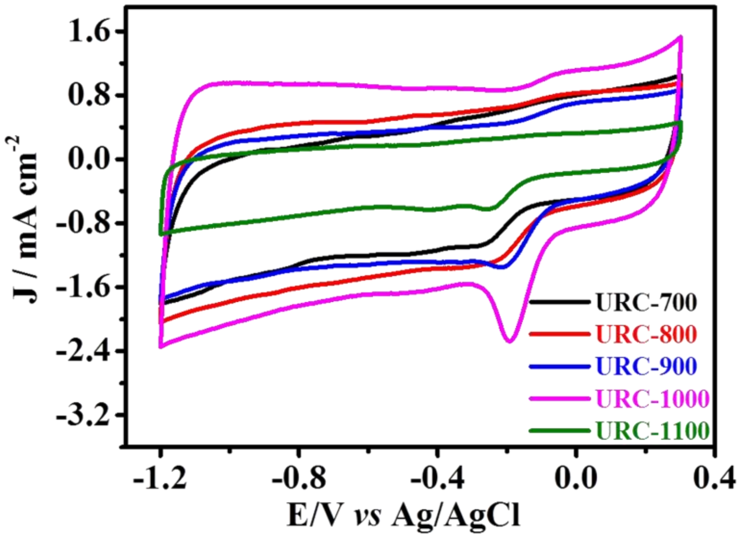

Figure S8 Cyclic voltammograms for URC materials electrodes in O_2 saturated 0.1 M KOH at 50 mV/s.

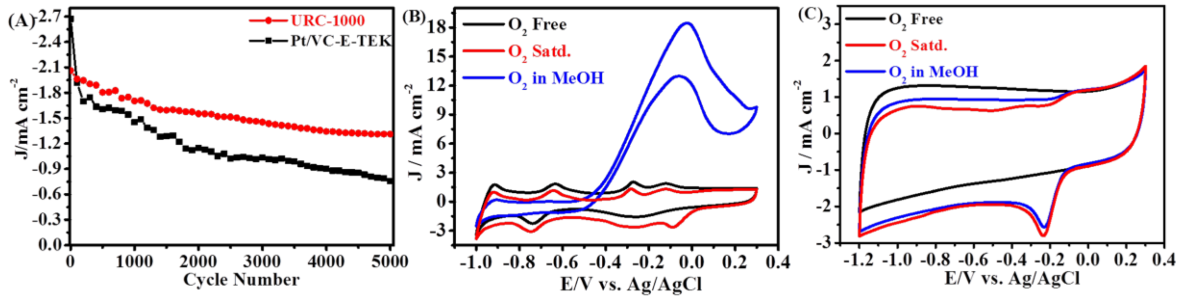

Figure S9 (A) ORR forward peak maximum current in CV for URC-1000 and 20 wt% Pt/C catalysts (E-TEK) during the repeated potential cycling up to 5000. Comparison of CV of 20 wt% Pt/C (B) and URC-1000 (C) electrodes for the ORR in O_2-saturated 0.1 M KOH with the presence and absence of methanol.

Resistivity measurement for the URC materials

To study the variation of electrical conductivity with pressure, a cell in four probe configuration was used for the measurement as shown in Figure S10. The cell was fabricated with some modification of an earlier report on measurement of electrical conductivity of powder samples (ref. S11). The cell consists of a hollow cylinder constructed with a non-conducting material (Teflon) in which two metallic pistons (Brass) form a pressure chamber. Current is applied to the sample through the metallic pistons and voltage is measured across the leads as shown in Figure S10. Keithley model 6220 and model 2182A were used as the DC current source and voltmeter, respectively. The current was varied from 0 to 10 mA, and the corresponding voltages were measured. The electrical conductivity of the samples were estimated using the formula shown in following equation (3)

$$\sigma = \frac{l}{RA} \tag{3}$$

where σ is the electrical conductivity, R is the resistance of the sample, A is the area of cross section of the sample (0.126 cm^2) and l is the distance between the voltage probes (0.2 cm). The pressure was varied by applying known weights on the metallic piston.

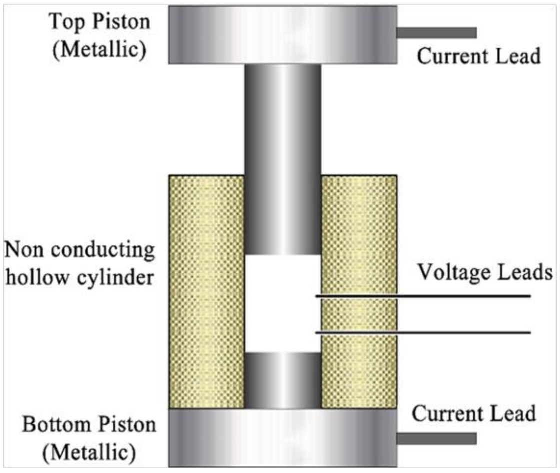

Figure S10 A diagram of the cell for measurement of electrical conductivity of powder samples.

Electrochemical impedance spectroscopy

To understand the electrocatalytic performances of the URC materials, electrochemical impedance spectroscopic (EIS) investigation was carried out and shown as Nyquist plots for URC-800 to URC-1100 materials in Figure S11A. The impedance diagrams are fit, considering the electrical equivalent circuits as shown in Figure S11B, and selected parameters are displayed in Table S3. This impedance data can be used to describe the interface properties. The charge transfer resistance (R_{ct}) values obtained for URC materials are 0.91, 0.57, 0.48, and 0.77 ohm for URC-800, URC-900, URC-1000, and URC-1100, respectively. The values of electrolyte resistance (R_s) are identical for all five URCs samples, suggesting similar compatibility with the electrolyte irrespective of the sample. This result indicates that the URC-1000 has a lower charge transfer resistance, which is more favorable for promoting both the electrocatalytic activity and the ORR kinetics than other URC materials. This can be attributed in part to high mesopore surface area and volume of URC-1000, which facilitates the O_2 and electrolyte movement toward the ORR active surface sites as summarized in Table 1. Thus, URC-1000 exhibits comparatively enhanced electrocatalytic activity as compared to the other URC materials, consequently proving to be the best electrode material for the ORR in terms of efficiency and kinetics.

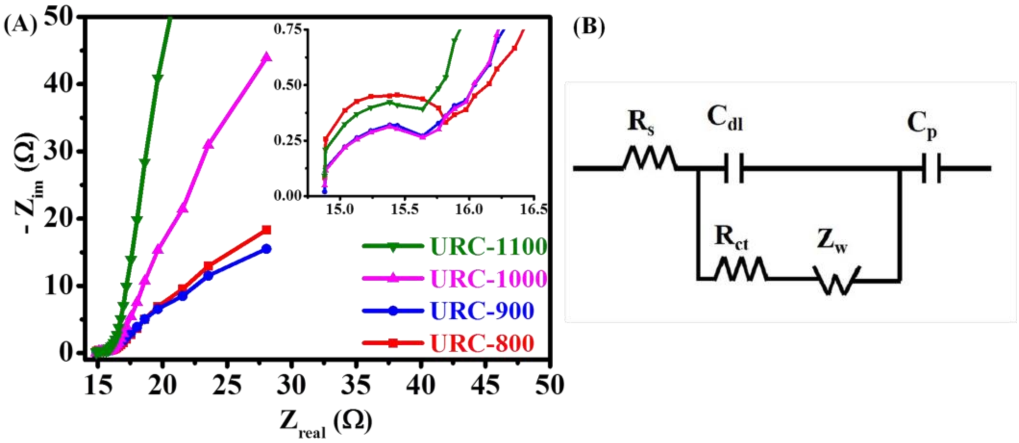

Figure S11 (A) Electrochemical impedance spectroscopic (EIS) Nyquist plots of the URC materials immersed in O_2-saturated 0.1 M KOH aqueous electrolytes. Inset: High frequency interval. (B) Corresponding electrical equivalent circuit.

Table S3. Electrochemical impedance parameter values extracted from the fit to the equivalent circuit for the URCs.

Sample Name	R_s (Ω)	R_{ct} (Ω)	C_{dl} (μF)	Z_w ($Ω.s^{-0.5}$)
URC-800	14.9	0.91	7.21	21.7
URC-900	14.8	0.57	14.28	30.6
URC-1000	14.9	0.48	17.15	26.1
URC-1100	14.9	0.77	8.55	40.1

References

S1. Lee, B. S. *et al.* Anodic properties of hollow carbon nanofibers for Li-ion battery. *J. Power Sources* **199**, 53-60 (2012).

S2. Wu, Z. S., Ren, W., Xu, L., Li, F. & Cheng, H. M. Doped graphene sheets as anode materials with superhigh rate and large capacity for lithium ion batteries. *ACS Nano* **5**, 5463-5471 (2011).

S3. Han, P. *et al.* Nitrogen-doping of chemically reduced mesocarbon microbead oxide for the improved performance of lithium ion batteries. *Carbon* **50**, 1355-1362 (2012).

S4. Paraknowitsch, J. P., Wienert, B., Zhang, Y., & Thomas, A. Intrinsically sulfur- and nitrogen-co-doped carbons from thiazolium salts. *Chem. Eur. J.* **18**, 15416-15423 (2012).

S5. Stankovich, S. *et al.* Synthesis of graphene-based nanosheets via chemical reduction of exfoliated graphite oxide. *Carbon* **45** 1558-1565 (2007).

S6. Wei, D. et al. Synthesis of N-doped graphene by chemical vapor deposition and its electrical properties. *Nano Lett.* **9**, 1752-1758 (2009).

S7. Maldonado, S. et al. Structure, composition, and chemical reactivity of carbon nanotubes by selective nitrogen doping. *Carbon* **44**, 1429-1437 (2006).

S8. Haerle, R., Riedo, E., Pasquarello, A., Baldereschi, A. sp^2/sp^3 hybridization ratio in amorphous carbon from C 1s core-level shifts: X-ray photoelectron spectroscopy and first-principles calculation. *Phys. Rev. B* **65,** 045101 (2001).

S9. Li, X. et al. Simultaneous nitrogen doping and reduction of graphene oxide. *J. Am. Chem. Soc.* **131**, 15939-15944 (2009).

S10. Sheng, Z.-H. *et al.* Catalyst-free synthesis of nitrogen-doped graphene via thermal annealing graphite oxide with melamine and its excellent electrocatalysis. *ACS Nano* **5**, 4350-4358 (2011).

S11. Espinola, A., Miguel, P. M., Salles, M. R. & Pinto, A. R. Electrical properties of carbons-resistance of powder materials. *Carbon* **24**, 337-341 (1986).
